# Yeast Two-Hybrid, a Powerful Tool for Systems Biology

**DOI:** 10.3390/ijms10062763

**Published:** 2009-06-18

**Authors:** Anna Brückner, Cécile Polge, Nicolas Lentze, Daniel Auerbach, Uwe Schlattner

**Affiliations:** 1 INSERM U884, Université Joseph Fourier, Laboratoire de Bioénergétique Fondamentale et Appliquée, 2280 Rue de la Piscine, BP 53, Grenoble Cedex 9, France; 2 Dualsystems Biotech AG / Grabenstrasse 11a, 8952 Schlieren, Switzerland

**Keywords:** interactomics, mass spectrometry, protein-protein interaction, systems bioenergetics, yeast two-hybrid

## Abstract

A key property of complex biological systems is the presence of interaction networks formed by its different components, primarily proteins. These are crucial for all levels of cellular function, including architecture, metabolism and signalling, as well as the availability of cellular energy. Very stable, but also rather transient and dynamic protein-protein interactions generate new system properties at the level of multiprotein complexes, cellular compartments or the entire cell. Thus, interactomics is expected to largely contribute to emerging fields like systems biology or systems bioenergetics. The more recent technological development of high-throughput methods for interactomics research will dramatically increase our knowledge of protein interaction networks. The two most frequently used methods are yeast two-hybrid (Y2H) screening, a well established genetic *in vivo* approach, and affinity purification of complexes followed by mass spectrometry analysis, an emerging biochemical *in vitro* technique. So far, a majority of published interactions have been detected using an Y2H screen. However, with the massive application of this method, also some limitations have become apparent. This review provides an overview on available yeast two-hybrid methods, in particular focusing on more recent approaches. These allow detection of protein interactions in their native environment, as e.g. in the cytosol or bound to a membrane, by using cytosolic signalling cascades or split protein constructs. Strengths and weaknesses of these genetic methods are discussed and some guidelines for verification of detected protein-protein interactions are provided.

## 1. Interactomics Take Center Stage in Systems Biology

### 1.1. A central role for protein interactions

The field of systems biology has achieved tremendous momentum during recent years. This development has been driven by: (i) a huge amount of genomic and proteomic data already available, (ii) the need to understand complex cellular systems or multifactorial diseases such as cancer or the metabolic syndrome, and (iii) emerging technologies which allow high-throughput screening of complex mixtures of biomolecules or non-invasive studies of live cells or entire organisms. In addition, evolution in this field would have been impossible without the parallel development of bioinformatics tools to analyze the large amounts of data generated.

Multiprotein complexes, not individual proteins, are increasingly recognized as the molecular basis of cellular fluxes of molecules, signals and energy. Thus, technologies which enable us to decipher cellular interactions between biomolecules (interactomics) together with those measuring metabolite fluxes (metabolomics, fluxomics) and signalling cascades (phosphoproteomics and others dealing with secondary protein modifications) have taken center stage in systems biology [1].

Interactomics can be applied in a global, unbiased cell systems approach, or in a more targeted approach to study a specific set of proteins [2]. While the former may identify so-called “nodes” or “hubs” in cell signalling but is often prone to errors (see discussion below on false negatives and positives), the latter is able to reliably describe sub-networks in more detail, including biophysical constants of the interaction and their spatiotemporal organization [3].

To date, the cellular interactome has mainly been explored for interactions involving proteins in the fields of cell signalling and cell architecture to understand the wiring of cellular data processing. However, it is also becoming increasingly important in many other fields.

### 1.2. Systems bioenergetics

Bioenergetics has known several decades of intense research, starting with the discovery of the main biochemical pathways and energy conservation in a chemiosmotic gradient in the 60s and 70s of the last century. After being a quiescent field for more than a decade, several developments during the last 15 years have put bioenergetics and mitochondria back to the forefront of scientific development [4] (for an excellent book see [5]): There has been the description of the involved protein machines at an atomic level (like the respiratory complexes in mitochondria), the discovery of a close link between mitochondria and cell signalling (calcium, apoptosis), and the emerging relationship between a dysfunction of cellular energetics and a plethora of complex pathologies, including (neuro-)muscular and age-related diseases, metabolic and cardiovascular diseases and cancer. Currently, the field of bioenergetics is about to enter the era of systems biology [6]. In fact, ATP generation needs a precise interplay between proteins of glycolysis, TCA cycle, mitochondrial electron transport and energy transfer systems like creatine kinase, which often includes specific (micro)compartmentation of proteins or multiprotein complexes maintained by specific protein-protein interactions. These topologies then allow for more precise regulation or have further thermodynamic advantages like substrate channelling between active sites. Systems bioenergetics holds the promise of integrating the multiple aspects of cellular energetics in a holistic approach which: (i) extends our knowledge on protein complexes involved in metabolic control and cell signalling [7], (ii) considers cellular compartmentation particularly important in this field [8], and (iii) aims to understand the complex regulatory cellular network which governs homeostasis in cell energetics and which apparently fails in so many pathologies [9]. Inversely, manipulating energy metabolism holds promises for therapeutic strategies. For example, it was surprising that inhibiting mitochondrial complex I in mitochondria is part of the molecular mechanism of the most successful antidiabetic drug, metformin [10]. Thus, the emerging field of systems bioenergetics does not only involve basic research, but is of prime importance for applied and clinical scientists.

For bioenergetics, interactomics goes far beyond cell signalling or cell structure, since it may uncover a new layer of regulation. The components of the mitochondrial redox chain or the ATPases are among the most complex protein assemblies, and understanding their regulation as well as the flux of protons and electrons will need intense work. Spatiotemporal organization of the long known pathways in primary metabolism is still incompletely understood [11], and the same applies to the systems of “energy-rich” intermediates like nucleoside triphosphates or phosphocreatine and mechanisms like metabolite channeling between different components in a complex [6,12,13].

### 1.3. Interactomics tools

This review gives an overview of several methods for global or targeted interactomics, with a particular emphasis on classical and emerging yeast two-hybrid (Y2H) systems. These Y2H tools now allow access to the almost entire cellular proteome for interaction screening, including membrane proteins, transcriptionally active proteins and proteins localized in different subcellular compartments. Massive application of such tools can be expected, since they are comparatively inexpensive as compared to others, do not need specialized large equipment and can be performed in any molecular biology laboratory with reasonable throughput.

### 2. Screening Technologies for Protein-Protein Interactions

Protein-protein interactions are involved in all cellular processes. Mapping of these interaction networks to elucidate the organization of the proteome into functional units is thus of prime importance for systems biology. A large number of methods have been developed for screening protein interactions. The more classical biochemical approaches, such as copurification, affinity purification or coimmunoprecipitation of protein complexes require *in vitro* handling of protein extracts. Further limitations of these techniques include restricted sensitivity and bias towards high affinity interactions. Once a partner has been detected, identification by mass spectrometry (MS) is generally straightforward, although rather costly. Cloning of corresponding cDNAs may be time-consuming, but clone repositories such as RIKEN or IMACE can be a convenient alternative. More recently, surface plasmon resonance (SPR), a biophysical technology, has been adopted for screening protein-protein interactions. Purified cellular extracts are injected onto a sensor chip covered with an immobilized binding partner. The instrument setup combines capture of the binding partner with a quantitative readout of the binding event, such that putative partners can be eluted and identified by MS [14,15]. Another approach to interaction screening are “cDNA-expression” libraries (for a review see [16]) such as phage display or Y2H methods, the latter detecting protein interactions *in vivo*. For studies on a genomic scale, highly parallel and automated processes are needed. However, only few detection methods for protein-protein interactions can be easily adapted for a high-throughput strategy. These include in particular yeast two-hybrid (Y2H) and affinity purification coupled to MS (AP/MS).

### 2.1. Yeast two-hybrid

The Y2H technique allows detection of interacting proteins in living yeast cells [17]. As described in full detail in chapter 3, interaction between two proteins, called bait and prey, activates reporter genes that enable growth on specific media or a color reaction. Y2H can be easily automated for high-throughput studies of protein interactions on a genome-wide scale, as shown for viruses like bacteriophage T7 [18], *Saccharomyces cerevisiae* [19,20], *Drosophila melanogaster* [21], *Caenorhabditis elegans* [22] and humans [23,24]. Experimental Y2H data have been a crucial part in establishing large synthetic human interactomes [25,26] or to dissect mechanisms in human disease [27]. Two screening approaches can be distinguished: the matrix (or array) and the library approach.

In the matrix approach, all possible combinations between full-length open reading frames (ORFs) are systematically examined by performing direct mating of a set of baits versus a set of preys expressed in different yeast mating types (e.g. mating type *a* for baits and mating type *α* for preys). This approach is easily automatable and has been used in yeast and human genome-scale two-hybrid screens. In yeast, 6,000 ORFs were cloned and over 5,600 interactions were identified, involving 70% of the yeast proteome [19,20,28]. The defined position of each bait in a matrix allows rapid identification of interacting preys without sequencing, but screens are usually restricted to a limited set of full length ORF’s and will thus fail to detect certain interactors (called false negatives).

The classical cDNA-library screen searches for pairwise interactions between defined proteins of interest (bait) and their interaction partners (preys) present in cDNA libraries or sub-pools of libraries. An exhaustive screen of libraries with selected baits can be an alternative to a matrix approach. Here, preys are not separated on an array but pooled (reviewed in [29]), and libraries may contain cDNA fragments in addition to full length ORFs, thus largely covering a transcriptome and reducing the rate of false negatives. However, inherent to this type of library screening, the rate of wrongly identified proteins (called false positives) is increased. In addition, interaction partners have to be identified by colony PCR analysis and sequencing, making such screens more expensive and time consuming.

### 2.2. Affinity purification/mass spectrometry

The value of MS for high-throughput screening of protein interactions has been recognized only more recently. This analytical technique is based on the determination of the mass-to-charge ratio of ionized molecules. Already introduced in 1948, sensitivity and implementation range of MS has been largely extended by technological advances. These include Nobel prize crowned methods for ionization like electrospray ionization [30], generating ions from macromolecules in liquid medium without their fragmentation, soft laser desorption (SLD) [31] or matrix-assisted laser desorption/ionization (MALDI) [32], using a laser beam for ionization of macromolecules without breaking chemical bonds. MS is now routinely applied to identify proteolytic fragments of proteins or even entire proteins and protein complexes [33]. Coupled to classic biochemical methods like affinity purification or chemical cross-linking, MS has become also a powerful tool for large-scale interactome research, mainly in form of affinity purification-MS (AP/MS). In this approach, a protein mostly fused to an epitope-tag is either immunoprecipitated by a specific antibody (e.g. against the tag) or purified by affinity columns recognizing the tag. Affinity purification can make use of an individual tag (e.g. a Flag-tag) for single step purification. However, it is more efficient when using two subsequent purification steps with proteins that are doubly tagged (e.g. 6xHis- and Strep-tag) or carry either C- or N-terminally a fusion of two affinity tags separated by a protease cleavage site (e.g. protein A and calmodulin binding protein) where the first tag is cleaved off after the first AP step (tandem affinity purification, TAP). This results in an enrichment of native multiprotein complexes containing the tagged protein. Subsequent MS analysis then identifies the different constituents of the complexes [34]. Ho *et al.* [35] expressed 10% of yeast ORFs with a C-terminal flag-tag under the control of an inducible promoter in yeast. They were able to connect 25% of the yeast proteome in a multiprotein complex interaction network. With the TAP-tag approach, Gavin *et al.* [36,37] and Krogan *et al.* [38] purified 1,993 and 2,357 TAP-fusion proteins covering 60% and 72% of the yeast proteome, respectively. As compared to the single Flag-tag approach, combination of two different purification steps in TAP results in improved sensitivity and specificity (TAP is reviewed in more detail in [39,40]). Recent technical progresses in automation of complex purification and MS analysis, together with dedicated computational methods to increase accuracy of data analysis, have made this approach a powerful tool in interactome research.

### 2.3. Comparison of Y2H- and MS-based methods

MS is less accessible than Y2H due to the expensive large equipment needed. Thus, a large amount of the data so far generated from protein interaction studies have come from Y2H screening. For example, more than 5,600 protein interactions have been so far reported for yeast [19,20,28,41] and about 6,000 for humans [23,24], establishing extensive protein interaction networks. Approximately half of the interaction data available on databases such as IntAct [42] and MINT [43] are coming from Y2H assays. Genome-scale Y2H screens have highlighted considerable cross-talk in the cell, even between proteins that were not thought to be functionally connected. However, Y2H and AP/MS are complementary in the kind of interactors that they are detecting. AP/MS may determine all the components of a larger complex, which not necessarily all interact directly with each other, while Y2H studies identify defined binary, interactions in this complex. In addition, some types of protein-protein interactions can be missed in Y2H due to inherent limitations, like interactions involving membrane proteins, self activating proteins, or proteins requiring post-translational modifications, but this may also occur with AP/MS-based approaches. Given the strengths of both methods, considerable effort is invested to overcome the remaining drawbacks. Different Y2H systems have been developed to extend the coverage of the proteome of interest, as will be described in detail further below. Recently, also the sensitivity and robustness of AP/MS was improved by the development of an integrated workflow coupling rapid generation of bait-expressing cell lines with an increase in protein complex purification using a novel double-affinity strategy [44]. Only a combination of different approaches that necessarily includes bioinformatics tools, will eventually lead to a fairly complete characterization of physiologically relevant protein-protein interactions in a given cell or organism. This will be a fundamental requirement to use interactome data in a systems biology approach at the cellular or higher complexity level.

## 3. Aiming at *in Vivo* Interactions: The Yeast Two-Hybrid Approach

### 3.1. Historical perspective: The principles of the approach

In 1989, Fields and Song revolutionized protein interaction analysis by describing a genetic system to detect direct protein-protein interactions in the yeast *Saccharomyces cerevisiae* [17]. Until then, interactions between two proteins had mostly been studied using biochemical techniques. The development of this completely new analytic tool was triggered by the molecular analysis of eukaryotic transcription factors. Only few years before, the Ptashne Laboratory had discovered the modular structure of Gal4, a transcriptional activator in yeast. They showed that Gal4 binds a specific DNA sequence (the upstream activation domain, UAS) and thus activates transcription in the presence of galactose. If separated into two fragments, the N-terminal fragment did still bind to DNA, but did not activate transcription in presence of galactose, while this latter function was mediated by the C-terminal fragment [45]. However, both fragments could interact and non-covalently reconstitute a fully functional Gal4. Thus, two different functional domains of Gal4 were identified: an N-terminal DNA binding domain (DBD) and a C-terminal (transcriptional) activation domain (AD), with both individual domains maintaining their function independent of the presence of the other.

**Figure 1.**
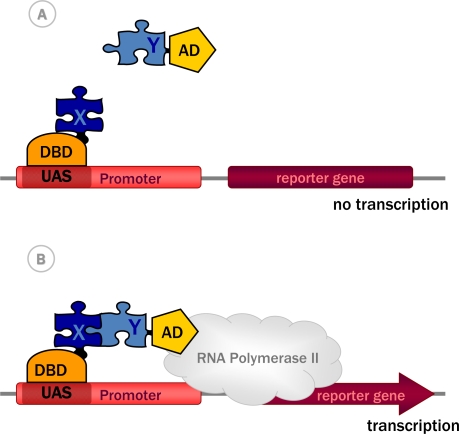
The classical yeast two-hybrid system. (A) The protein of interest X, is fused to the DNA binding domain (DBD), a construct called bait. The potential interacting protein Y is fused to the activation domain (AD) and is called prey. (B) The bait, i.e. the DBD-X fusion protein, binds the upstream activator sequence (UAS) of the promoter. The interaction of bait with prey, i.e. the AD-Y fusion protein, recruits the AD and thus reconstitutes a functional transcription factor, leading to further recruitment of RNA polymerase II and subsequent transcription of a reporter gene.

Inspired by these findings, Fields and Song exploited the modular properties of the transcription factor Gal4 to monitor protein-protein interactions. The basic idea was to fuse the two proteins of interest X and Y to DBD and AD of Gal4, respectively, such that interaction between X and Y reconstitutes a functional transcription factor that could then drive reporter gene expression (Figure 1). In the first construct called bait, protein X (e.g. the glucose-sensor SNF1) was fused to the N-terminal part of GAL4 containing the DBD (GAL4DBD). In the second construct, the prey, protein Y (e.g. the regulatory protein SNF4) was fused to the C-terminal part of Gal4 that contains the AD (GAL4AD). Expression of both fusion proteins in yeast and interaction between bait and prey indeed reconstituted a functional Gal4 transcription factor from the two separate polypeptides. Gal4 then recruited RNA polymerase II, leading to transcription of a *GAL1-lacZ* fusion gene. This reporter gene encodes the enzyme beta-galactosidase which labels the yeast cell when using a colorimetric substrate [17].

For a genome-wide screen for interactors of given baits, a cDNA library is used to construct an entire library of preys. From a methodological point of view, any such Y2H screen implies the transformation of yeast cells with bait and prey cDNA on different vectors under the control of yeast promoters. Expression levels will depend on the promoter used and may affect sensitivity and specificity of the screen. Once expressed in the cytosol, bait and prey must be able to enter the nucleus to activate transcription, a limitation of the original Y2H approach further discussed below.

This classical Y2H system has been extended to exploit different other DNA-binding proteins (e.g. DBD of *E. coli* repressor protein LexA), transcriptional activators (e.g. AD of Herpes simplex virus VP16) and various reporter genes. A suitable reporter gene must encode a protein whose function provides a simple readout. Thus, besides the colorimetric reaction with the *lacZ* gene, the most commonly used are auxotrophic markers (e.g. *LEU2, HIS3, ADE2, URA3, LYS2*) that allow growth on minimal media. In the current state-of-the-art, more than one reporter gene is assayed in parallel to increase the stringency of Y2H screens [46]. In fact, one of the common problems of Y2H is the generation of false positives due to non-specific interactions (as described in detail further below). Selection for two active reporter genes requires a more solid transcriptional activation and thus increases the stringency of the assay, but concomitantly penalizes detection of weak and transient interactions. Another possibility to adjust the stringency of the assay is partial inhibition of the enzymatic activity encoded by the reporter gene. For example, the product of the *HIS3* reporter, imidazole glycerol phosphate dehydratase, is competitively inhibited by increasing concentrations of 3-aminotriazole.

Compared to earlier interaction screens, the Y2H system was able to detect interactions *in vivo* in a true cellular environment. Since it is also relatively easy to implement and inexpensive, Y2H rapidly became the system of choice for detecting protein-protein interactions. Its principles were rapidly adopted for screenings involving interaction of more than two partners. To analyse ligand-receptor interactions, a synthetic heterodimer of two different small organic ligands is used as a third hybrid molecule together with two receptors fused to DBD and AD. In this case, binding of the hybrid organic ligand to both receptors will force them together to reconstitute the DBD-AD complex [47]. This three hybrid system can also be used to identify inhibitors of protein-protein interactions [48]. Another extension of the classical Y2H system is the use of more than one bait, in particular to compare interaction specificities [49]. In the so-called Dual Bait system, protein X_1_ is fused to the LexA DBD, and protein X_2_ is fused to the DBD of the cI repressor from bacteriophage λ. Thus, each bait is directed to a different reporter gene. Positive interactions with X_1_ are registered through lexA operator activation of *LEU2* and *LacZ*, and positive interactions with X_2_ through cI operator activation of *LYS2* and *GusA. GusA* codes for beta-glucuronidase that can use a colorimetric substrate to report interactions. This system has been successfully used to identify proteins interacting with specific regions in larger proteins [50]. Further more recent expansions of Y2H to high-throughput applications, the so-called matrix or array approach, has been already discussed in the previous chapter.

In their original publication Fields and Song already mentioned some of the limits of their Y2H method: “The system requires that the interaction can occur within the yeast nucleus, that the Gal4-activating region is accessible to the transcription machinery and that the Gal4(1-147)-protein X hybrid is itself not a potent activator”. These limitations would exclude almost half of all proteins, explaining the great interest for developing alternative Y2H variants.

### 3.2. Choosing the right strategy: Available Y2H systems and their advantages

**Figure 2.**
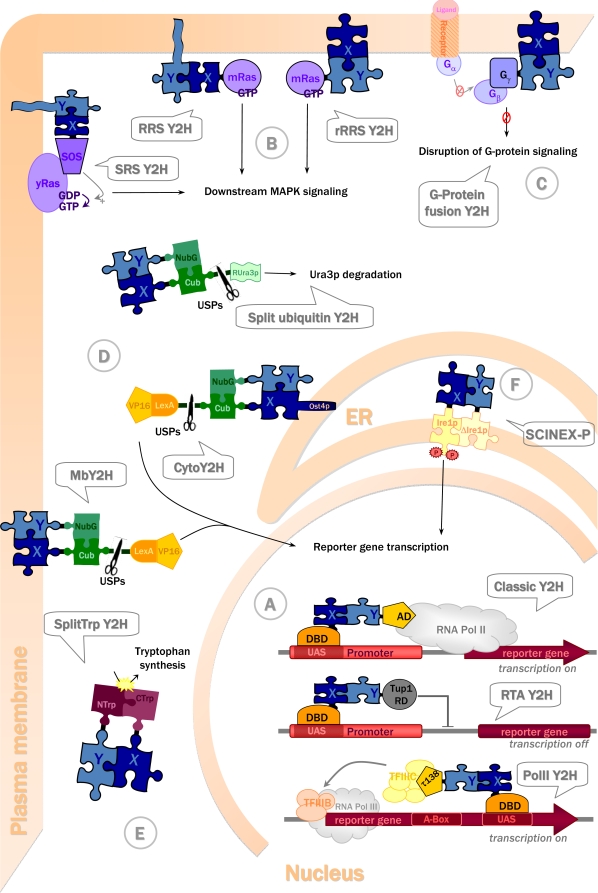
Yeast two-hybrid systems, their subcellular location within a yeast cell, and their operating mode (represented at the moment of bait-prey interaction). Protein X (dark blue puzzle piece, part of bait construct) and protein Y (light blue puzzle piece, part of prey construct) directly interact (fitting puzzle pieces), thus inducing reconstitution of split-proteins (puzzle pieces of different colors in A, D, E), membrane recruitment (B, C) or protein dimerization (F). Protein fusions in bait or prey constructs are shown as solid black lines between puzzle pieces. Bait-prey interaction activates further downstream events (arrows) that directly (A) or indirectly (B, C, D, F) lead to transcriptional activation, or are independent of transcriptional activation (D, E), finally yielding screenable readouts like growth on specific media or color reactions. (A) ***Nuclear Y2H systems*** all require protein recruitment and bait-prey interaction at nuclear DNA. The classic Y2H and RTA Y2H both engage RNA polymerase II (RNA Pol II) transcription either by its activation or its inhibition. By contrast, the Pol III Y2H, involves RNA polymerase III (RNA Pol III) transcription. (B) ***Ras signalling based Y2H at the plasma membrane.*** The SRS Y2H, RRS Y2H, and rRRS Y2H are all based on protein recruitment to the plasma membrane via bait-prey interaction and subsequent activation of MAPK downstream signalling. While in the SRS and RRS Y2H the prey constructs harboring protein Y are anchored at the membrane via myristoylation to analyze interactions with cytosolic bait constructs harboring protein X, the rRRS is used to analyze interactions between soluble preys containing protein Y and partner X being a membrane protein. (C) ***G-protein signalling-based Y2H at the plasma membrane.*** In the G-protein fusion Y2H, bait X is a membrane or membrane-associated protein whose interaction with the prey construct disrupts protein G downstream signalling. (D) ***Split-ubiquitin based Y2H systems*** involve reconstitution of ubiquitin from two domains upon bait-prey interaction. Their subcellular localization depends on the nature of interacting proteins X or Y, and on the reporter proteins used. The Split ubiquitin Y2H uses non-transcriptional reporting of protein interactions in the cytosol, but can also be used for membrane proteins (not shown). The MbY2H is used for interaction analysis with membrane baits and thus occurs at the membrane location of protein X, e.g. the plasma membrane. The CytoY2H is used for membrane anchored cytosolic baits and occurs close to the ER membrane (E) ***Split-protein sensor Y2H.*** The Split-Trp Y2H is used to assay cytosolic bait-prey interactions based on reconstitution of an enzyme in tryptophan synthesis, allowing for non-transcriptional reporting. (F) ***ER Y2H system.*** The SCINEX-P Y2H allows bait-prey interaction analysis in the reducing environment of the ER, based on protein dimerization in unfolded protein signalling. ER, endoplasmic reticulum; for further abbreviations and details see chapter 3.2.

More recent Y2H-based techniques access almost the entire cellular proteome (see Table 1). Almost all of them rely on a similar principle, namely the modular structure of the protein reporting the interaction. Similar to DBD and AD reconstituting a transcription factor in the original Y2H system, they employ proteins containing two structural domains which can fold correctly independently of each other and which reconstitute the functional reporter system if brought together via bait-prey interaction. An exception of this principle is the recruitment-based Y2H, where the reporter cascade is activated by forced membrane localization of the bait-prey complex. The following chapter will present in more detail the currently available Y2H systems (Table 1, Figure 2).

#### 3.2.1. Y2H with transactivating proteins in the nucleus

The classic Y2H system is based on reconstitution of a transcription factor and thus not adapted for interaction analysis with proteins that can directly activate transcription. Such transactive baits would trigger transcription in absence of any interaction with a prey. Two alternative Y2H systems have been developed to analyze the interaction network of such proteins. One is based on repression of transactivation, while the other uses the alternative polymerase III transcription pathway. Also methods mentioned under 3.2.2 (e.g. the split-ubiquitin systems) are suitable to screen transactive baits.

In the **repressed transactivator (RTA) system** (Figure 2A), inversely to the classic Y2H, the bait-prey interaction represses transcriptional activation of reporter genes [60]. The protein of interest X fused to the DBD of Gal4 is transactive, e.g. a transcription factor. If it interacts with another protein Y fused to the repression domain (RD) of a transcription repressor (e.g. Tup1p), the transcription of the reporter gene is repressed [60]. The RTA system has been used to demonstrate interactions between the mammalian basic helix-loop-helix proteins MyoD and E12, and between the protooncogenic transcription factor c-Myc and the putative tumor suppressor protein Bin1 [60]. It has also been applied to screen for novel interactions with a variety of transcriptional activators, including herpes simplex virus 1 (HSV-1) regulatory protein VP16 [60], c-Myc [62], and the androgen receptor [61].

Recently, this system has been extended to screen for molecules which inhibit protein-protein interaction, for example between the immunophilin FKBP12 and the transforming growth factor β receptor (TGFβ-R) C terminus [67]. FKBP12 itself is not transactivating, but was fused to VP16-AD in addition to Gal4-DBD. In the absence of interaction with a RD-fusion protein, e.g. due to the presence of an inhibitor, transcription of reporter gene *HIS3* is activated. Strength of the inhibition is translated into expression levels of *HIS3* which can be probed by increasing amounts of 3-aminotriazole, a competitive inhibitor of the *HIS3* gene product. Compared to the classic Y2H, this assay has the advantage that inhibition of interaction does not result in a loss but in a gain of reporter gene transcription and thus in a positive signal facilitating screening procedures. Thus, the RTA Y2H can not only be used to identify interaction partners of transcription factors, but also as a reversed Y2H to screen small molecule libraries e.g. for potentially novel therapeutic compounds acting as inhibitors of a given protein-protein interaction.

The **RNA polymerase III based two-hybrid (Pol III) system** (Figure 2A) is another alternative to screen for interaction partners of transcription factors activating RNA polymerase II-based transcription. As in the classic Y2H, a protein X is fused to a Gal4-DBD (bait), and this bait is able to bind DNA due to Gal4p binding sequence artificially introduced into the reporter gene *SNR6*. However, the prey construct is different, since the second protein Y is fused to τ138p. This protein is a subunit of the multimeric protein complex TFIIIC, one of the two transcription factors involved in RNA polymerase III (PolIII)-mediated transcription. If now the bait interacts with the prey containing τ138p, the TFIIIC complex is bound to DNA and recruits a second transcription factor (TFIIIB) and Pol III. This will activate transcription of the *SNR6* reporter gene to produce U6 snRNA [68]. In a yeast strain harboring a temperature-sensitive U6 snRNA mutant [59], this reporter gene transcription will rescue the temperature-sensitive phenotype and allow yeast growth at 37°C. The system has been used to screen a mouse embryonic cDNA library using τ138p-mBRCA1 as a bait [59], but apparently has not been further adopted for screening assays.

#### 3.2.2. Y2H with cytosolic and membrane proteins

The classic Y2H and the two alternative systems presented above require the translocation of the interacting proteins into the nucleus and are thus not suitable for membrane associated proteins, integral membrane proteins and many other soluble cytosolic proteins or proteins localized in other subcellular compartments. To circumvent these limitations, truncated versions of these proteins have been used for Y2H screens [69–71]. However, the use of such truncated proteins can lead to misfolding, and the problem remains that the nucleus is not the natural environment for most of these proteins. Such problems, probably leading to a high rate of false negatives in the past, would be circumvented by screening procedures where interacting proteins remain in their natural cellular compartment. Outside the nucleus, away from the transcription machinery, also the use of transactivating baits would no longer constitute a problem.

The **SOS- and the RAS recruitment systems (SRS and RRS)** (Figure 2B) are bypassing the transcriptional readout by using the Ras signalling pathway, which is homologous between yeast and mammals. Ras has to be localized at the plasma membrane to undergo GDP-GTP exchange by guanyl exchange factors, Cdc25 in yeast or Son of sevenless (SOS) in mammals. This activated Ras then triggers downstream signalling. For the Y2H systems described here, a Cdc25-2 temperature sensitive yeast strain is used which is unable to grow at a higher temperature (36°C) because Cdc25-2 becomes inactive and fails to activate Ras signalling. The temperature-sensitive phenotype can then be rescued by alternative activation of Ras in the Y2H setup.

In the SOS recruitment system (Figure 2B: SRS Y2H), a soluble protein X is fused to mammalian SOS. If the SOS-X fusion interacts with a prey localized in the membrane (e.g. via myristoylation), SOS stimulates guanyl exchange on yeast Ras (yRas) and promotes downstream signalling [51].

In the Ras recruitment system (Figure 2B: RRS Y2H), the soluble protein X is directly fused to constitutively active mammalian Ras (mRas). Already active, this Ras only requires membrane location, bypassing the activity of Ras guanyl exchange factors (Cdc25 or SOS). The mRas-X fusion is recruited to the membrane by interaction with a membrane associated prey [57].

Both SRS and RRS allow the analysis of interactions between soluble baits and soluble or membrane preys. Specifically for the use of membrane localized baits, the reverse Ras recruitment system (Figure 2B: rRRS Y2H) has been developed. Conversely to the RRS, the prey is the Ras fusion protein, and the bait is membrane-anchored or itself a membrane protein [63]. Although the rRRs has been used for screening procedures [63], it has an important disadvantage. Preys containing membrane proteins are self-activating, since they localize Ras to the membrane even without bait-prey interaction. These false positive membrane proteins have to be eliminated by additional selection steps, rendering the method more laborious. Exclusion of membrane and membrane-associated proteins also represents serious limitation as compared to other more recent Y2H techniques.

The **G-protein fusion system** (Figure 2C) allows, similar to the rRRS, to study the interaction between integral membrane bait and a soluble prey. The latter is a fusion protein with the γ-subunit of a heterotrimeric G-protein. If the prey interacts with the membrane-located bait, it will sequester G-protein β-subunits, thus disrupting formation of heterotrimeric G-protein complex and subsequent downstream signalling [58]. The method has been used to identify neuronal Sec1 mutants unable to bind syntaxin1, a member of the SNARE complex [58]. Similar as with the RTA Y2H system (see above, Figure 2A), the authors suggest that G-protein Y2H may identify drugs disrupting protein-protein interactions. Both systems report disrupted interaction by a gain of signal which is easier to detect in a library screen as compared to a loss of signal.

The **Split-ubiquitin system** (Figure 2D) was designed by Johnsson and Varshavsky in 1994 [53] to allow detection of protein-protein interactions occurring between cytosolic proteins; it was later extended to membrane proteins. Ubiquitin is a small protein important for the turnover of cellular proteins. Proteins are labelled for proteasomal degradation by covalently attaching a poly-ubiquitin chain. This chain is then cleaved off prior to protein degradation by ubiquitin specific proteases (USP). The split ubiquitin Y2H technique is based on separation of ubiquitin into two independent fragments. It has been shown that ubiquitin can be split into an N-terminal (Nub) and a C-terminal half (Cub) and that these two parts retain a basic affinity for each other, thus allowing spontaneous reassembly of quasi-native ubiquitin. This spontaneous reassociation of Nub and Cub is abolished by point mutations (I13G or I13A) in Nub (NubG, NubA) [53]. In these mutants, efficient association is only observed if the two moieties are brought into close proximity by interaction of two proteins fused to NubG/A and Cub respectively. Reconstituted split-ubiquitin is recognized by USPs, which then cleave off any reporter protein fused to the C-terminal end of Cub. The original system used dihydrofolate reductase as reporter protein, whose release was detected by SDS-PAGE [53]. However, this readout was not convenient, since it needed immunoprecipitation and electrophoretic separation.

Looking for a more direct readout, Ura3p protein has been used as reporter (Figure 2D: Split ubiquitin Y2H) [72]. Ura3p is an orotidine 5-phosphate decarboxylase (ODCase), an enzyme involved in the synthesis of pyrimidine ribonucleotides. ODCase activity leads to uracil auxotrophy and sensitivity to 5-fluoroorotic acid (5-FOA), because the latter is converted into the toxic compound 5-fluorouracil, causing cell death. As Y2H reporter, a variant of Ura3p is used, rUra3p, which is N-terminally modified for rapid degradation according to the N-end rule [73]. Interaction between bait and prey leads to ubiquitin reconstitution and subsequent cleavage of rUra3p, resulting in rapid degradation of rUra3p, inability to grow on minimal medium without uracil, and resistance to 5-FOA. This system is not based on a transcriptional readout and can therefore be applied to nuclear, cytoplasmic and membrane proteins [74–76].

In the membrane transactivator split-ubiqitin (MbY2H) system, an artificial transcription factor (LexA-VP16) has been used as a cleavable reporter protein to analyse interactions between membrane proteins of the endoplasmic reticulum (ER) (Figure 2D: MbY2H) [55]. Once ubiquitin is reassembled, LexA-VP16 is released to the nucleus, where it activates reporter gene transcription (i.e. *HIS3*, *LacZ*). Such a transcriptional readout leads to an amplification of the response following protein interactions and offers more sensitivity, more convenient for transient interactions. This system was successfully used to detect interactions involving different kinds of membrane proteins [56]. Split-ubiquitin based systems have become quite popular and have been successfully applied for cDNA library screens [77–81] and large scale matrix approaches [82].

Recently, an adaptation of the MbY2H system to screen cytosolic proteins has been published (Figure 2D: CytoY2H) [66]. Here, the bait construct contains both Cub and the transcription factor and is anchored to the ER membrane thanks to a fusion to the ER membrane protein Ost4p. This allows screening for interaction partners of a soluble protein among membrane and/or soluble proteins, as well as for proteins that are transcriptional activators or otherwise self-activating in nuclear Y2H.

**Other Split-protein sensors** (Figure 2E) have been developed, inspired by the split-ubiquitin system. While the cytosolic Y2H methods presented above are based on indirect readout that requires activation of signalling pathways or transcription, split-protein sensors can in principle also directly report their reconstitution. In 2004, Tafelmeyer *et al.* presented a combinatorial approach to generate split-protein sensors [65]. They used an enzyme in yeast tryptophan biosynthesis, N-(5-phosphoribosyl)-anthranilate isomerase (Trp1p), to perform activity selections of different combinations of fragment pairs. They identified C-terminal (CTrp) and N-terminal (NTrp) fragments which reconstitute a quasi-native Trp1p only when fused to two interacting proteins that bring the CTrp and NTrp domains into close proximity. Thus, interacting fragments lead to Trp1p reconstitution and allow *trp1* deficient yeast strains to grow on medium lacking tryptophan (Figure 2E: SplitTrpY2H). This system has several advantages. The readout is direct and permutation-independent, i.e. independent of whether CTrp or NTrp were used for bait constructs. It is universally applicable to all types of proteins, because the interaction readout is entirely independent of cellular localization.

Recently, split enhanced green fluorescent protein has been used to monitor protein-protein interactions in yeast by confocal microscopy [53]. A variety of other split-protein sensors has been applied in eukaryotic cells (e.g. dihydrofolate reductase [54], β-galactosidase [55], β-lactamase [56]), but has not yet been used in Y2H screening.

#### 3.2.3. Yeast two-hybrid with extracellular and transmembrane proteins

All Y2H systems presented so far detect interactions in the reducing intracellular environment, which is not necessarily ideal for extracellular proteins. However, interactions in the extracellular space, like between receptors and ligands or between antibodies and antigens, participate in a multitude of physiological processes, and their study is of particular interest for a better understanding of numerous pathologies.

The **SCINEX-P (screening for interactions between extracellular proteins) system** (Figure 2F) published by Urech *et al.* in 2003 allows the analysis of protein-protein interactions in the oxidizing environment of the ER [64]. This system exploits the signalling of the yeast unfolded protein response (UPR). Accumulation of incorrectly folded proteins in the ER induces dimerization of the yeast ER type I transmembrane protein (Ire1p), which induces production of transcriptional activator Hac1p that will activate transcription of chaperons. In the SCINEX-P system, proteins of interest are fused to mutated Ire1p proteins, one lacking its luminal, N-terminal oligomerization domain (ΔIre1p). The interaction between two hybrid proteins then reconstitutes Ire1p dimerization and thus activates UPR downstream signalling. To monitor protein interactions, the Hac1p UPR element is introduced into the promoter of reporter genes. This Y2H system was successfully used to analyze the interaction between the protein disulfide isomerase ERp57 and Calnexin, both involved in protein folding in the ER [83], as well as known interactions between antigens and antibodies [64].

#### 3.3. Dealing with doubt: Limitations of Y2H systems and methods for its validation

Its relative methodical simplicity, its diversity, and its high-throughput capacity make the Y2H system the most popular analytic and screening method for interactomics. Nevertheless, all Y2H methods face the problem of false negatives and false positives.

**False negatives** in Y2H are protein-protein interactions which cannot be detected due to limitations of the screening method. In the classic Y2H, for example, protein interactions involving membrane proteins are mostly undetectable. Thus, the Y2H strategy has to be chosen carefully, depending on the cellular sub-proteome of interest. Further, the interaction between the two proteins assayed in Y2H is often not symmetric (permutation-independent), meaning it depends on whether a given protein is used for fusion in the bait or the prey construct. The fused yeast reporter proteins or anchors may cause steric hindrance that impedes interaction, thus causing false negatives. Another reason for false negatives can be different or lacking post-translational protein modifications in the yeast system when analyzing interactions between proteins of higher eukaryotes. In this case, the modifying enzyme may be coexpressed in yeast together with bait and prey. This possibility has been used with success to identify tyrosine-phosphorylation dependent interactions [84]. Very transient interactions may also escape detection, as e.g. in case of substrate interactions of protein tyrosine phosphatase. Here, substrate-trap mutants have been used lacking phosphatase activity but retaining their affinity for the substrates to identify protein substrates of the phosphatase [85]. The expression of baits fused to their cognate modifying enzyme has been successfully used to identify acetylation dependent interactions with histones and interactions dependent on phosphorylation of the carboxy-terminal domain of RNA polymerase II [86]. The lack of more complex modifications, like complex glycosylation, appears to be more difficult to overcome. A humanized yeast strain has already been used to produce human glycosylated proteins in yeast *Pichia pastoris* [87], but it has so far not been used in Y2H.

False negatives mainly cause problems in reproducibility of Y2H screens. Two independent large-scale Y2H screens using the same Y2H method showed less than 30% overlap in the identified interactions and only 12,5% of known interactions were found in each of both [19]. These discrepancies may arise from a difference in selection stringency or a difference in the cDNA library used. Thus, false negatives represent a real limitation of the Y2H system in representing an entire protein interaction network. However, each screening system has to deal with false negatives. For example, MS of purified protein complexes reveals only few interactions involving transmembrane proteins due to their difficult purification [88]. AP/MS was also shown to be biased towards highly abundant proteins, whereas protein abundance appears not to influence Y2H [88]. While purification of protein complexes has to deal with mixtures of proteins showing very different abundance, depending on the used cell type, such differences are avoided in Y2H by overexpression of interacting proteins at similar levels. However, protein overexpression can provoke other artefacts such as false positives.

**False positives** in Y2H are physical interactions detected in the screening in yeast which are not reproducible in an independent system. They are of diverse origin and often depend on the Y2H system used. Among possible reasons for false positive interactions in yeast may be a high expression level of bait and prey and their localization in a compartment which does not correspond to their natural cellular environment. Another source of false positives is interaction of prey with the reporter proteins (e.g. LexA in the classic Y2H) or the membrane anchors (e.g. Ost4p in the cytoY2H) fused to the bait. Proteins which allow yeast to overcome nutritional selection when overexpressed are also often scored as false positives. Finally, proteins that are known to be “sticky” or that are not correctly folded can show unspecific interactions. In general, for each Y2H system, a list of recurrent false positives can be established. A list created by Golemis and co-workers for the classic Y2H can be found at http://www.fccc.edu/research/labs/golemis/InteractionTrapInWork.html.

Despite these limitations, the Y2H system remains a powerful tool for large-scale screening in interactomics. The comparative assessment of high-throughput screening methods by von Mehring *et al.* [88] revealed that Y2H has a lower coverage of the protein interaction network than the purification of protein complexes coupled to MS. But these authors only considered the classic Y2H, while the above presented diversity of Y2H systems may increase coverage considerably.

To evaluate the quality of a generated interaction data set, coverage and accuracy need to be considered together. In fact, a large interaction network cannot be a solid base for systems biology if confidence in the data is low. In a quantitative comparison of interaction data sets, von Mehring estimated the accuracy of a classic high-throughput Y2H screen to be less than 10%. Thus, the question remains how to increase accuracy of Y2H interaction data sets.

As mentioned before, there are two different screening approaches: the targeted library screening approach and the global matrix screening approach. To increase accuracy of a library screen, a bait-dependency test can be performed [66,94]. In this case, the previously identified preys are tested for interaction with unrelated baits. Preys interacting with others than the screening bait will be classified as false positives. This test helps to eliminate false positives resulting from non-specific interactions with the bait or other “sticky” interactions overcoming nutritional selection, but it cannot eliminate physical interactions, artificially occurring in the Y2H system without physiological meaning. For this reason, binary interactions detected in Y2H are nowadays published only if they are validated by other methods [80,89–91,93]. Different validation methods that can be used are listed in Table 2.

It is advisable to use more than one method to validate an identified protein-protein interaction, preferentially coupling biochemical methods (pull down assay, immunoprecipitation, Biacore surface plasmon resonance) with *in vivo/in situ* methods (colocalization, immunohistochemistry, *in situ* hybridization). The former methods allow the study of physical protein interactions, but pull-down assays require a certain stability of the protein complex or, in case of Biacore, even need purified interaction partners. It may be also difficult to validate transient protein interactions or protein interactions with transmembrane proteins in these assays. The *in vivo/in situ* methods allow insight into possible coexpression and colocalization of the two proteins involved, but generally do not provide conclusive evidence for direct interaction. However, an advantage of *in situ* hybridization would be its adaptability for high-throughput. The FRET method has been developed to go beyond protein colocalization *in vivo* to study the spatio-temporal occurrence of the interaction and its physiological significance. FRET can only occur when the distance separating the two different fluorophores is in the low nm-range, a condition that occurs if fluorophores are coupled to two directly interacting proteins [95]. However, many of these methods are relatively labor intensive and can only be applied to a small number of interactions detected in a larger screen.

Validation of results from high-throughput matrix studies is much more difficult to achieve. Using the mentioned validation methods would be experimentally extremely difficult. Given that both interaction partners are randomly selected, the large amount of generated interaction data would already render a bait dependency test impossible. To handle the problem of false positives in such large-scale approaches, help is coming from computational biology. Confidence scores can evaluate the biological significance and probability of a given interaction. One possibility is to relate screening results to known data like RNA expression levels (expression profile reliability (EPR) index), or interaction networks of protein paralogues (paraloguous verification method (PVM)) [96]. Another score was calculated by combining data on sequence homology, known interacting Pfam domains and Gene Ontology annotations [97]. Even if these methods allow creation of higher confidence scores, they are limited by the number of existing data from other screens and experiments. Another possibility is thus the creation of a statistical model only based on screen data and topological criteria [98]. These scores will not replace experimental validation of detected interactions, but may provide a tool to select proteins for further experiments.

## 4. Further Confirmation: Protein-Protein Interactions within a Biological System

Once a protein-protein interaction has been identified and validated, the physiological function of a given interaction remains to be established in a biological system. The main questions in this respect are: (i) Where and when in the system the interaction does occur? (ii) Which parameters influence the interaction? (iii) What is the effect of the interaction? To answer these questions, the main strategy relies on varying different system parameters that mainly affects the proteins of interest. Combination of a panel of complementary methods is generally able to unveil the physiological significance of an interaction identified in a targeted approach.

Colocalization experiments in cell culture under different conditions can give information about the spatiotemporal dynamics of the protein-protein interactions. For example, choosing different time points during the cell-cycle may reveal transient colocalizations. In the case of the reported interaction between brain type creatine kinase (BCK) and the cis-Golgi matrix protein (GM130), a transient colocalization during early prophase was observed [91]. The authors suggest that BCK would facilitate GM130 phosphorylation by ATP-requiring protein kinases and thus play a role in initial fragmentation of the Golgi apparatus prior to cell division. Many other endogenous or external parameters influencing protein-protein interaction can be varied, including activation of signalling cascades or changes in the cellular environment. To analyse the impact of given protein-protein interactions on the cellular phenotype, the interaction may be either disturbed, e.g. by RNA silencing of one interaction partner, or favoured by addition or overexpression of one protein partner. More specifically, the interaction domains of both interaction partners can be mapped to inhibit the interaction *in vivo* by expressing interaction-deficient mutant proteins or using inhibitory peptides.

These experiments can be carried out for defined interactions of a small number of proteins, but again it would be quite difficult to transfer them to the large interaction network generated by global screens. So far, interactome approaches concentrate on a characterization of the nodes in the interaction network, which may be the major determinants of a phenotype.

## 5. Conclusions

Since systems biology aims at a complete representation of cellular complexity, thus avoiding any reductionism, the applied experimental strategies have to provide non-biased, complete data sets. In this context, the yeast two-hybrid technologies presented here are a starting point rather than a complete solution to the elucidation of interaction networks. However, Y2H has demonstrated its power by its methodological diversity and technical simplicity to rapidly generate a large amount of reliable protein-protein interaction data. More recent Y2H technologies, in particular those based on split proteins, allow to probe protein-protein interactions in their native cellular compartment and to access almost the entire cellular proteome. Y2H is rather complementary in respect to emerging AP/MS techniques, since it identifies direct interactions and also detects interaction of lower affinity that are rather transient.

Developing high throughput approaches at the cellular level and further progress in bioinformatics will be necessary to make interactomics a fully integral part of a systems biology approach. Major efforts will be necessary for the challenge of modelling the large and dynamic interaction network of a cell. Only a combination of different approaches (e.g. Y2H, MS, bioinformatics) will eventually lead to an accurate description of large interaction networks.

## Figures and Tables

**Table 1.** Overview of different Y2H systems and their specificities.

**Year**	**Y2H method**	**Possible baits**	**Response**	Cellular compartment *	**Screen compatibility #**
1989	Classic Y2H system [17]	Non-transactivating proteins capable of entering nucleus	Transcriptional activation	Nucleus	Yes [17]
1994	SOS recruitment system (SRS) [51]	Transactivating, cytosolic proteins	Ras signalling	Membrane periphery	Yes [52]
1994	Split-ubiquitin system [53]	Nuclear, membrane and cytosolic proteins	Uracil auxotrophy and 5-FoA resistance	Cytosol	Yes [54]
1998	Membrane split-ubiquitin system (MbY2H) [55]	Membrane proteins	Transcriptional activation	Membrane periphery	Yes [56]
1998	Ras recruitment system (RRS) [57]	Transactivating, cytosolic proteins	Ras signalling	Membrane periphery	Yes [57]
1999	Dual bait system [49]	Two non-transactivating proteins capable of entering nucleus	Transcriptional activation	Nucleus	Yes [49]
2000	G-protein fusion system [58]	Membrane proteins	Inhibition of protein G signalling	Membrane periphery	No
2001	RNA polymerase III based two-hybrid (Pol III) [59]	Transactivating proteins (in the RNA polymerase II pathway)	Transcriptional activation	Nucleus	Yes [59]
2001	Repressed transactivator system (RTA) [60]	Transactivating proteins capable of entering nucleus	Inhibition of transcriptional activation	Nucleus	Yes [60–62]
2001	Reverse Ras recruitment system (rRRS) [63]	Membrane proteins	Ras signalling	Membrane periphery	Yes [63]
2003	SCINEX-P system [64]	Extracellular and transmembrane proteins	Downstream signalling & transcriptional activation	Endoplasmic reticulum (ER)	No
2004	Split-Trp system [65]	Cytosolic, membrane proteins	Trp1p activity	Cytosol	Yes (Lentze & Auerbach, unpubl.)
2007	Cytosolic split-ubiquitin system (cytoY2H) [66]	Transactivating, cytosolic proteins	Transcriptional activation	ER membrane periphery	Yes [66]

* Cellular compartment where the interaction occurs.

^#^ Indicates whether a given Y2H system has been used for cDNA-library screening.

**Table 2.** Overview of different validation methods.

**Method**	**Type**	**Description**
Pull-down assay [89–91]	*in vitro*	Tagged bait (mostly expressed in *E.coli*) is immobilized on a resin and subsequently “pulls down” target protein (prey) from lysates (of eukaryotic cells or of *E.coli* expressing proteins of interest). After washing steps, prey is detected by SDS-PAGE/immunoblot or MS.
Coimmunoprecipitation [80,89,90,92]	*ex vivo*	A specific antibody is used to precipitate the bait from cell lysates (see above). After washing steps, coimmunoprecipitated prey is detected as above.
Surface plasmon resonance (Biacore) [93]	*in vitro*	Bait immobilized on the surface of a sensor chip is probed by injection of prey onto the surface. Protein interaction is detected online via a biophysical principle (using the change in refractive index at the sensor surface in case of protein interaction). Protein is eluted and analyzed by MS.
*In situ* hybridization [90]	*in situ*	Hybridization of a labelled complementary DNA or RNA strand (i.e. probe) to a specific DNA or RNA sequence in a tissue section. Visualizes expression of specific genes to evaluate potential coexpression of proteins of interest in the same cell of a given tissue.
Immunohistochemistry, immunocytochemistry [80,89,90]	*in situ*	Proteins in fixed cells or tissue sections are detected by immune-labelling with fluorescently tagged antibodies, e.g. using confocal microscopy. Visualizes coexpression of proteins of interest in the same cell and potential subcellular colocalization.
Fluorescent detection in live cells [91]	*in vivo*	Proteins in living cells are detected with fluorescently tagged antibodies as above (using permeabilized cells) or after expression of fluorescently tagged protein variants. Visualizes colocalization of proteins of interest.
Fluorescence resonance energy transfer (FRET) [80]	*in vivo*	Bait and prey are fused to two different fluorescent tags with overlapping emission/excitation spectra. If both proteins are in close proximity, excitation of the first fluorophore (donor) leads to energy transfer to the second fluorophore (acceptor). Acceptor fluorescence can be observed *in vitro* (fluorimeter) or in living cells (confocal microscopy).
Bioluminescencer resonance energy transfer (BRET) [92]	*in vivo*	Similar to FRET (see above), but with bait fused to bioluminescent luciferase, thus avoiding the external excitation step susceptible to generate background. Detection as with FRET.

## References

[1] Kiemer L Cesareni G Comparative interactomics: comparing apples and pears? Trends Biotechnol 2007 25 448 454 17825444 10.1016/j.tibtech.2007.08.002

[2] Kelly W Stumpf M Protein-protein interactions: from global to local analyses Curr. Opin. Biotechnol 2008 19 396 403 18644446 10.1016/j.copbio.2008.06.010

[3] Charbonnier S Gallego O Gavin AC The social network of a cell: recent advances in interactome mapping Biotechnol. Annu. Rev 2008 14 1 28 18606358 10.1016/S1387-2656(08)00001-X

[4] Scheffler IE Mitochondria make a come back Adv. Drug. Deliv. Rev 2001 49 3 26 11377800 10.1016/s0169-409x(01)00123-5

[5] Scheffler IE Mitochondria2nd ed John Wiley & Sons Hoboken, New Jersey, USA 2007

[6] Saks V Kaambre T Guzun R Anmann T Sikk P Schlattner U Wallimann T Aliev M Vendelin M The creatine kinase phosphotransfer network: thermodynamic and kinetic considerations, the impact of the mitochondrial outer membrane and modelling approaches Subcell Biochem 2007 46 27 65 18652071 10.1007/978-1-4020-6486-9_3

[7] Bruggeman FJ Westerhoff HV The nature of systems biology Trends Microbiol 2007 15 45 50 17113776 10.1016/j.tim.2006.11.003

[8] Srere PA Knull HR Location-location-location Trends Biochem. Sci 1998 23 319 320 9787634 10.1016/s0968-0004(98)01262-6

[9] Beal MF Mitochondria take center stage in aging and neurodegeneration Ann. Neurol 2005 58 495 505 16178023 10.1002/ana.20624

[10] Guigas B Detaille D Chauvin C Batandier C De Oliveira F Fontaine E Leverve X Metformin inhibits mitochondrial permeability transition and cell death: a pharmacological *in vitro* study Biochem. J 2004 382 877 884 15175014 10.1042/BJ20040885 PMC1133963

[11] Ovadi J Srere PA Macromolecular compartmentation and channeling Int. Rev. Cytol 2000 192 255 280 10553282 10.1016/s0074-7696(08)60529-x

[12] Schlattner U Gehring F Vernoux N Tokarska-Schlattner M Neumann D Marcillat O Vial C Wallimann T C-terminal lysines determine phospholipid interaction of sarcomeric mitochondrial creatine kinase J. Biol. Chem 2004 279 24334 24342 15044463 10.1074/jbc.M314158200

[13] Schlattner U Wallimann T Metabolite channeling: creatine kinase microcompartments Encyclopedia of Biological Chemistry Lennarz WJ Lane MD Academic Press New York, NY, USA 2004 646 651

[14] Boireau W Rouleau A Lucchi G Ducoroy P Revisited BIA-MS combination: entire “on-a-chip” processing leading to the proteins identification at low femtomole to sub-femtomole levels Biosens. Bioelectron 2009 24 1121 1127 18829299 10.1016/j.bios.2008.06.030

[15] Natsume T Nakayama H Jansson O Isobe T Takio K Mikoshiba K Combination of biomolecular interaction analysis and mass spectrometric amino acid sequencing Anal. Chem 2000 72 4193 4198 10994983 10.1021/ac000167a

[16] Phizicky EM Fields S Protein-protein interactions: methods for detection and analysis Microbiol. Rev 1995 59 94 123 7708014 10.1128/mr.59.1.94-123.1995 PMC239356

[17] Fields S Song O A novel genetic system to detect protein-protein interactions Nature 1989 340 245 246 2547163 10.1038/340245a0

[18] Bartel PL Roecklein JA SenGupta D Fields S A protein linkage map of Escherichia coli bacteriophage T7 Nat. Genet 1996 12 72 77 8528255 10.1038/ng0196-72

[19] Ito T Chiba T Ozawa R Yoshida M Hattori M Sakaki Y A comprehensive two-hybrid analysis to explore the yeast protein interactome Proc. Natl. Acad. Sci. USA 2001 98 4569 4574 11283351 10.1073/pnas.061034498 PMC31875

[20] Uetz P Giot L Cagney G Mansfield TA Judson RS Knight JR Lockshon D Narayan V Srinivasan M Pochart P Qureshi-Emili A Li Y Godwin B Conover D Kalbfleisch T Vijayadamodar G Yang M Johnston M Fields S Rothberg JM A comprehensive analysis of protein-protein interactions in Saccharomyces cerevisiae Nature 2000 403 623 627 10688190 10.1038/35001009

[21] Formstecher E Aresta S Collura V Hamburger A Meil A Trehin A Reverdy C Betin V Maire S Brun C Jacq B Arpin M Bellaiche Y Bellusci S Benaroch P Bornens M Chanet R Chavrier P Delattre O Doye V Fehon R Faye G Galli T Girault JA Goud B de Gunzburg J Johannes L Junier MP Mirouse V Mukherjee A Papadopoulo D Perez F Plessis A Rosse C Saule S Stoppa-Lyonnet D Vincent A White M Legrain P Wojcik J Camonis J Daviet L Protein interaction mapping: a Drosophila case study Genome Res 2005 15 376 384 15710747 10.1101/gr.2659105 PMC551564

[22] Obrdlik P El-Bakkoury M Hamacher T Cappellaro C Vilarino C Fleischer C Ellerbrok H Kamuzinzi R Ledent V Blaudez D Sanders D Revuelta JL Boles E Andre B Frommer WB K^+^ channel interactions detected by a genetic system optimized for systematic studies of membrane protein interactions Proc. Natl. Acad. Sci. USA 2004 101 12242 12247 15299147 10.1073/pnas.0404467101 PMC514463

[23] Stelzl U Worm U Lalowski M Haenig C Brembeck FH Goehler H Stroedicke M Zenkner M Schoenherr A Koeppen S Timm J Mintzlaff S Abraham C Bock N Kietzmann S Goedde A Toksoz E Droege A Krobitsch S Korn B Birchmeier W Lehrach H Wanker EE A human protein-protein interaction network: a resource for annotating the proteome Cell 2005 122 957 968 16169070 10.1016/j.cell.2005.08.029

[24] Rual JF Venkatesan K Hao T Hirozane-Kishikawa T Dricot A Li N Berriz GF Gibbons FD Dreze M Ayivi-Guedehoussou N Klitgord N Simon C Boxem M Milstein S Rosenberg J Goldberg DS Zhang LV Wong SL Franklin G Li S Albala JS Lim J Fraughton C Llamosas E Cevik S Bex C Lamesch P Sikorski RS Vandenhaute J Zoghbi HY Smolyar A Bosak S Sequerra R Doucette–Stamm L Cusick ME Hill DE Roth FP Vidal M Towards a proteome-scale map of the human protein-protein interaction network Nature 2005 437 1173 1178 16189514 10.1038/nature04209

[25] Rhodes DR Tomlins SA Varambally S Mahavisno V Barrette T Kalyana-Sundaram S Ghosh D Pandey A Chinnaiyan AM Probabilistic model of the human protein-protein interaction network Nat. Biotechnol 2005 23 951 959 16082366 10.1038/nbt1103

[26] Gandhi TK Zhong J Mathivanan S Karthick L Chandrika KN Mohan SS Sharma S Pinkert S Nagaraju S Periaswamy B Mishra G Nandakumar K Shen B Deshpande N Nayak R Sarker M Boeke JD Parmigiani G Schultz J Bader JS Pandey A Analysis of the human protein interactome and comparison with yeast, worm and fly interaction datasets Nat. Genet 2006 38 285 293 16501559 10.1038/ng1747

[27] Lim J Hao T Shaw C Patel AJ Szabo G Rual JF Fisk CJ Li N Smolyar A Hill DE Barabasi AL Vidal M Zoghbi HY A protein-protein interaction network for human inherited ataxias and disorders of Purkinje cell degeneration Cell 2006 125 801 814 16713569 10.1016/j.cell.2006.03.032

[28] Fromont-Racine M Mayes AE Brunet-Simon A Rain JC Colley A Dix I Decourty L Joly N Ricard F Beggs JD Legrain P Genome-wide protein interaction screens reveal functional networks involving Sm-like proteins Yeast 2000 17 95 110 10900456 10.1002/1097-0061(20000630)17:2<95::AID-YEA16>3.0.CO;2-H PMC2448332

[29] Auerbach D Thaminy S Hottiger MO Stagljar I The post-genomic era of interactive proteomics: facts and perspectives Proteomics 2002 2 611 623 12112840 10.1002/1615-9861(200206)2:6<611::AID-PROT611>3.0.CO;2-Y

[30] Fenn JB Mann M Meng CK Wong SF Whitehouse CM Electrospray ionization for mass spectrometry of large biomolecules Science 1989 246 64 71 2675315 10.1126/science.2675315

[31] Tanaka K Waki H Ido Y Akita S Yoshida Y Yoshida T Protein and polymer analyses up to m/z 100 000 by laser ionization time-of-flight mass spectrometry Rapid Commun. Mass Spectrom 1988 2 151 153

[32] Karas M Hillenkamp F Laser desorption ionisation of proteins with molecular masses exceeding 10.000 daltons Anal. Chem 1988 60 2299 2301 3239801 10.1021/ac00171a028

[33] Nazabal A Wenzel RJ Zenobi R Immunoassays with direct mass spectrometric detection Anal. Chem 2006 78 3562 3570 16737208 10.1021/ac0519108

[34] Pandey A Mann M Proteomics to study genes and genomes Nature 2000 405 837 46 10866210 10.1038/35015709

[35] Ho Y Gruhler A Heilbut A Bader GD Moore L Adams SL Millar A Taylor P Bennett K Boutilier K Yang L Wolting C Donaldson I Schandorff S Shewnarane J Vo M Taggart J Goudreault M Muskat B Alfarano C Dewar D Lin Z Michalickova K Willems AR Sassi H Nielsen PA Rasmussen KJ Andersen JR Johansen LE Hansen LH Jespersen H Podtelejnikov A Nielsen E Crawford J Poulsen V Sorensen BD Matthiesen J Hendrickson RC Gleeson F Pawson T Moran MF Durocher D Mann M Hogue CW Figeys D Tyers M Systematic identification of protein complexes in Saccharomyces cerevisiae by mass spectrometry Nature 2002 415 180 183 11805837 10.1038/415180a

[36] Gavin AC Bosche M Krause R Grandi P Marzioch M Bauer A Schultz J Rick JM Michon AM Cruciat CM Remor M Hofert C Schelder M Brajenovic M Ruffner H Merino A Klein K Hudak M Dickson D Rudi T Gnau V Bauch A Bastuck S Huhse B Leutwein C Heurtier MA Copley RR Edelmann A Querfurth E Rybin V Drewes G Raida M Bouwmeester T Bork P Seraphin B Kuster B Neubauer G Superti-Furga G Functional organization of the yeast proteome by systematic analysis of protein complexes Nature 2002 415 141 147 11805826 10.1038/415141a

[37] Gavin AC Aloy P Grandi P Krause R Boesche M Marzioch M Rau C Jensen LJ Bastuck S Dumpelfeld B Edelmann A Heurtier MA Hoffman V Hoefert C Klein K Hudak M Michon AM Schelder M Schirle M Remor M Rudi T Hooper S Bauer A Bouwmeester T Casari G Drewes G Neubauer G Rick JM Kuster B Bork P Russell RB Superti-Furga G Proteome survey reveals modularity of the yeast cell machinery Nature 2006 440 631 636 16429126 10.1038/nature04532

[38] Krogan NJ Cagney G Yu H Zhong G Guo X Ignatchenko A Li J Pu S Datta N Tikuisis AP Punna T Peregrin-Alvarez JM Shales M Zhang X Davey M Robinson MD Paccanaro A Bray JE Sheung A Beattie B Richards DP Canadien V Lalev A Mena F Wong P Starostine A Canete MM Vlasblom J Wu S Orsi C Collins SR Chandran S Haw R Rilstone JJ Gandi K Thompson NJ Musso G St Onge P Ghanny S Lam MH Butland G Altaf-Ul AM Kanaya S Shilatifard A O'Shea E Weissman JS Ingles CJ Hughes TR Parkinson J Gerstein M Wodak SJ Emili A Greenblatt JF Global landscape of protein complexes in the yeast Saccharomyces cerevisiae Nature 2006 440 637 643 16554755 10.1038/nature04670

[39] Collins MO Choudhary JS Mapping multiprotein complexes by affinity purification and mass spectrometry Curr. Opin. Biotechnol 2008 19 324 330 18598764 10.1016/j.copbio.2008.06.002

[40] Puig O Caspary F Rigaut G Rutz B Bouveret E Bragado-Nilsson E Wilm M Seraphin B The tandem affinity purification (TAP) method: a general procedure of protein complex purification Methods 2001 24 218 229 11403571 10.1006/meth.2001.1183

[41] Uetz P Hughes RE Systematic and large-scale two-hybrid screens Curr. Opin. Microbiol 2000 3 303 308 10851163 10.1016/s1369-5274(00)00094-1

[42] Hermjakob H Montecchi-Palazzi L Lewington C Mudali S Kerrien S Orchard S Vingron M Roechert B Roepstorff P Valencia A Margalit H Armstrong J Bairoch A Cesareni G Sherman D Apweiler R IntAct: an open source molecular interaction database Nucleic Acids Res 2004 32Database issueD452 D455 14681455 10.1093/nar/gkh052 PMC308786

[43] Zanzoni A Montecchi-Palazzi L Quondam M Ausiello G Helmer-Citterich M Cesareni G MINT: a Molecular INTeraction database FEBS Lett 2002 513 135 140 11911893 10.1016/s0014-5793(01)03293-8

[44] Glatter T Wepf A Aebersold R Gstaiger M An integrated workflow for charting the human interaction proteome: insights into the PP2A system Mol. Syst. Biol 2009 5 237 19156129 10.1038/msb.2008.75 PMC2644174

[45] Keegan L Gill G Ptashne M Separation of DNA binding from the transcription-activating function of a eukaryotic regulatory protein Science 1986 231 699 704 3080805 10.1126/science.3080805

[46] Durfee T Becherer K Chen PL Yeh SH Yang Y Kilburn AE Lee WH Elledge SJ The retinoblastoma protein associates with the protein phosphatase type 1 catalytic subunit Genes Dev 1993 7 555 569 8384581 10.1101/gad.7.4.555

[47] Licitra EJ Liu JO A three-hybrid system for detecting small ligand-protein receptor interactions Pro.c Natl. Acad. Sci. USA 1996 93 12817 12821 10.1073/pnas.93.23.12817 PMC24003 8917502

[48] Huang J Schreiber SL A yeast genetic system for selecting small molecule inhibitors of protein-protein interactions in nanodroplets Proc. Natl. Acad. Sci. USA 1997 94 13396 13401 9391035 10.1073/pnas.94.25.13396 PMC28315

[49] Serebriiskii I Khazak V Golemis EA A two-hybrid dual bait system to discriminate specificity of protein interactions J. Biol. Chem 1999 274 17080 17087 10358061 10.1074/jbc.274.24.17080

[50] Serebriiskii IG Mitina O Pugacheva EN Benevolenskaya E Kotova E Toby GG Khazak V Kaelin WG Chernoff J Golemis EA Detection of peptides, proteins, and drugs that selectively interact with protein targets Genome Res 2002 12 1785 1791 12421766 10.1101/gr.450702 PMC187545

[51] Aronheim A Engelberg D Li N al-Alawi N Schlessinger J Karin M Membrane targeting of the nucleotide exchange factor Sos is sufficient for activating the Ras signaling pathway Cell 1994 78 949 961 7923364 10.1016/0092-8674(94)90271-2

[52] Aronheim A Zandi E Hennemann H Elledge SJ Karin M Isolation of an AP-1 repressor by a novel method for detecting protein-protein interactions Mol Cell Biol 1997 17 3094 3102 9154808 10.1128/mcb.17.6.3094 PMC232162

[53] Johnsson N Varshavsky A Split ubiquitin as a sensor of protein interactions *in vivo* Proc. Natl. Acad. Sci. USA 1994 91 10340 10344 7937952 10.1073/pnas.91.22.10340 PMC45015

[54] Dirnberger D Messerschmid M Baumeister R An optimized split-ubiquitin cDNA-library screening system to identify novel interactors of the human Frizzled 1 receptor Nucleic Acids Res 2008 36 e37 18319286 10.1093/nar/gkm1163 PMC2330230

[55] Stagljar I Korostensky C Johnsson N te Heesen S A genetic system based on split-ubiquitin for the analysis of interactions between membrane proteins *in vivo* Proc. Natl. Acad. Sci. USA 1998 95 5187 5192 9560251 10.1073/pnas.95.9.5187 PMC20236

[56] Hooker BS Bigelow DJ Lin CT Methods for mapping of interaction networks involving membrane proteins Biochem. Biophys. Res. Commun 2007 363 457 461 17897627 10.1016/j.bbrc.2007.09.031

[57] Broder YC Katz S Aronheim A The ras recruitment system, a novel approach to the study of protein-protein interactions Curr. Biol 1998 8 1121 1124 9778531 10.1016/s0960-9822(98)70467-1

[58] Ehrhard KN Jacoby JJ Fu XY Jahn R Dohlman HG Use of G-protein fusions to monitor integral membrane protein-protein interactions in yeast Nat. Biotechnol 2000 18 1075 1079 11017046 10.1038/80274

[59] Petrascheck M Castagna F Barberis A Two-hybrid selection assay to identify proteins interacting with polymerase II transcription factors and regulators Biotechniques 2001 30 296 298300, 302. 11233598 10.2144/01302st02

[60] Hirst M Ho C Sabourin L Rudnicki M Penn L Sadowski I A two-hybrid system for transactivator bait proteins Proc. Natl. Acad. Sci. USA 2001 98 8726 8731 11447261 10.1073/pnas.141413598 PMC37503

[61] Huang A Ho CS Ponzielli R Barsyte-Lovejoy D Bouffet E Picard D Hawkins CE Penn LZ Identification of a novel c-Myc protein interactor, JPO2, with transforming activity in medulloblastoma cells Cancer Res 2005 65 5607 5619 15994933 10.1158/0008-5472.CAN-05-0500

[62] Wafa LA Cheng H Rao MA Nelson CC Cox M Hirst M Sadowski I Rennie PS Isolation and identification of L-dopa decarboxylase as a protein that binds to and enhances transcriptional activity of the androgen receptor using the repressed transactivator yeast two-hybrid system Biochem. J 2003 375 373 383 12864730 10.1042/BJ20030689 PMC1223690

[63] Hubsman M Yudkovsky G Aronheim A A novel approach for the identification of protein-protein interaction with integral membrane proteins Nucleic Acids Res 2001 29 e18 11160938 10.1093/nar/29.4.e18 PMC29625

[64] Urech DM Lichtlen P Barberis A Cell growth selection system to detect extracellular and transmembrane protein interactions Biochim. Biophys. Acta 2003 1622 117 127 12880949 10.1016/s0304-4165(03)00133-8

[65] Tafelmeyer P Johnsson N Johnsson K Transforming a (beta/alpha)8--barrel enzyme into a split-protein sensor through directed evolution Chem. Biol 2004 11 681 689 15157879 10.1016/j.chembiol.2004.02.026

[66] Mockli N Deplazes A Hassa PO Zhang Z Peter M Hottiger MO Stagljar I Auerbach D Yeast split-ubiquitin-based cytosolic screening system to detect interactions between transcriptionally active proteins Biotechniques 2007 42 725 730 17612295 10.2144/000112455

[67] Joshi PB Hirst M Malcolm T Parent J Mitchell D Lund K Sadowski I Identification of protein interaction antagonists using the repressed transactivator two-hybrid system Biotechniques 2007 42 635 644 17515203 10.2144/000112434

[68] Marsolier MC Prioleau MN Sentenac A A RNA polymerase III-based two-hybrid system to study RNA polymerase II transcriptional regulators J. Mol. Biol 1997 268 243 249 9159467 10.1006/jmbi.1997.0979

[69] Borg JP Marchetto S Le Bivic A Ollendorff V Jaulin-Bastard F Saito H Fournier E Adelaide J Margolis B Birnbaum D ERBIN: a basolateral PDZ protein that interacts with the mammalian ERBB2/HER2 receptor Nat. Cell Biol 2000 2 407 414 10878805 10.1038/35017038

[70] Sugita S Hata Y Sudhof TC Distinct Ca(2+)-dependent properties of the first and second C2-domains of synaptotagmin I J. Biol. Chem 1996 271 1262 1265 8576108 10.1074/jbc.271.3.1262

[71] Niethammer M Kim E Sheng M Interaction between the C terminus of NMDA receptor subunits and multiple members of the PSD-95 family of membrane-associated guanylate kinases J. Neurosci 1996 16 2157 2163 8601796 10.1523/JNEUROSCI.16-07-02157.1996 PMC6578538

[72] Wittke S Lewke N Muller S Johnsson N Probing the molecular environment of membrane proteins *in vivo* Mol. Biol. Cell 1999 10 2519 2530 10436009 10.1091/mbc.10.8.2519 PMC25484

[73] Varshavsky A The N-end rule pathway of protein degradation Genes Cells 1997 2 13 28 9112437 10.1046/j.1365-2443.1997.1020301.x

[74] Laser H Bongards C Schuller J Heck S Johnsson N Lehming N A new screen for protein interactions reveals that the Saccharomyces cerevisiae high mobility group proteins Nhp6A/B are involved in the regulation of the GAL1 promoter Proc. Natl. Acad. Sci. USA 2000 97 13732 13737 11095729 10.1073/pnas.250400997 PMC17644

[75] Kerkmann K Lehming N Genome-wide expression analysis of a Saccharomyces cerevisiae strain deleted for the Tup1p-interacting protein Cdc73p Curr. Genet 2001 39 284 290 11525400 10.1007/s002940100229

[76] Eckert JH Johnsson N Pex10p links the ubiquitin conjugating enzyme Pex4p to the protein import machinery of the peroxisome J. Cell. Sci 2003 116 3623 3634 12876220 10.1242/jcs.00678

[77] Thaminy S Auerbach D Arnoldo A Stagljar I Identification of novel ErbB3-interacting factors using the split-ubiquitin membrane yeast two-hybrid system Genome Res 2003 13 1744 1753 12840049 10.1101/gr.1276503 PMC403748

[78] Wang B Pelletier J Massaad MJ Herscovics A Shore GC The yeast split-ubiquitin membrane protein two-hybrid screen identifies BAP31 as a regulator of the turnover of endoplasmic reticulum-associated protein tyrosine phosphatase-like B Mol. Cell. Biol 2004 24 2767 2778 15024066 10.1128/MCB.24.7.2767-2778.2004 PMC371098

[79] Matsuda S Giliberto L Matsuda Y Davies P McGowan E Pickford F Ghiso J Frangione B D'Adamio L The familial dementia BRI2 gene binds the Alzheimer gene amyloid-beta precursor protein and inhibits amyloid-beta production J. Biol. Chem 2005 280 28912 28916 15983050 10.1074/jbc.C500217200

[80] Felkl M Leube RE Interaction assays in yeast and cultured cells confirm known and identify novel partners of the synaptic vesicle protein synaptophysin Neuroscience 2008 156 344 352 18706977 10.1016/j.neuroscience.2008.07.033

[81] Pasch JC Nickelsen J Schunemann D The yeast split-ubiquitin system to study chloroplast membrane protein interactions Appl. Microbiol. Biotechnol 2005 69 440 447 15988575 10.1007/s00253-005-0029-3

[82] Miller JP Lo RS Ben-Hur A Desmarais C Stagljar I Noble WS Fields S Large-scale identification of yeast integral membrane protein interactions Proc. Natl. Acad. Sci. USA 2005 102 12123 12128 16093310 10.1073/pnas.0505482102 PMC1189342

[83] Pollock S Kozlov G Pelletier MF Trempe JF Jansen G Sitnikov D Bergeron JJ Gehring K Ekiel I Thomas DY Specific interaction of ERp57 and calnexin determined by NMR spectroscopy and an ER two-hybrid system Embo. J 2004 23 1020 1029 14988724 10.1038/sj.emboj.7600119 PMC380975

[84] Osborne MA Zenner G Lubinus M Zhang X Songyang Z Cantley LC Majerus P Burn P Kochan JP The inositol 5′-phosphatase SHIP binds to immunoreceptor signaling motifs and responds to high affinity IgE receptor aggregation J. Biol. Chem 1996 271 29271 29278 8910587 10.1074/jbc.271.46.29271

[85] Fukada M Kawachi H Fujikawa A Noda M Yeast substrate-trapping system for isolating substrates of protein tyrosine phosphatases: Isolation of substrates for protein tyrosine phosphatase receptor type z Methods 2005 35 54 63 15588986 10.1016/j.ymeth.2004.07.008

[86] Guo D Hazbun TR Xu XJ Ng SL Fields S Kuo MH A tethered catalysis, two-hybrid system to identify protein-protein interactions requiring post-translational modifications Nat. Biotechnol 2004 22 888 892 15208639 10.1038/nbt985

[87] Hamilton SR Gerngross TU Glycosylation engineering in yeast: the advent of fully humanized yeast Curr. Opin. Biotechnol 2007 18 387 392 17951046 10.1016/j.copbio.2007.09.001

[88] von Mering C Krause R Snel B Cornell M Oliver SG Fields S Bork P Comparative assessment of large-scale data sets of protein-protein interactions Nature 2002 417 399 403 12000970 10.1038/nature750

[89] Jackson M Song W Liu MY Jin L Dykes-Hoberg M Lin CI Bowers WJ Federoff HJ Sternweis PC Rothstein JD Modulation of the neuronal glutamate transporter EAAT4 by two interacting proteins Nature 2001 410 89 93 11242047 10.1038/35065091

[90] Tanaka H Katoh H Negishi M Pragmin, a novel effector of Rnd2 GTPase, stimulates RhoA activity J. Biol. Chem 2006 281 10355 10364 16481321 10.1074/jbc.M511314200

[91] Burklen TS Hirschy A Wallimann T Brain-type creatine kinase BB-CK interacts with the Golgi Matrix Protein GM130 in early prophase Mol. Cell Biochem 2007 297 53 64 17036164 10.1007/s11010-006-9322-4

[92] Dye DE Karlen S Rohrbach B Staub O Braathen LR Eidne KA Coombe DR hShroom1 links a membrane bound protein to the actin cytoskeleton Cell. Mol. Life Sci 2009 66 681 696 19137261 10.1007/s00018-009-8645-1 PMC11131465

[93] Hornemann T Kempa S Himmel M Hayess K Furst DO Wallimann T Muscle-type creatine kinase interacts with central domains of the M-band proteins myomesin and M-protein J. Mol. Biol 2003 332 877 887 12972258 10.1016/s0022-2836(03)00921-5

[94] Fetchko M Stagljar I Application of the split-ubiquitin membrane yeast two-hybrid system to investigate membrane protein interactions Methods 2004 32 349 362 15003597 10.1016/j.ymeth.2003.10.010

[95] Day RN Visualization of Pit-1 transcription factor interactions in the living cell nucleus by fluorescence resonance energy transfer microscopy Mol. Endocrinol 1998 12 1410 1419 9731708 10.1210/mend.12.9.0168

[96] Deane CM Salwinski L Xenarios I Eisenberg D Protein interactions: two methods for assessment of the reliability of high throughput observations Mol. Cell Proteomics 2002 1 349 356 12118076 10.1074/mcp.m100037-mcp200

[97] Patil A Nakamura H Filtering high-throughput protein-protein interaction data using a combination of genomic features BMC Bioinformatics 2005 6 100 15833142 10.1186/1471-2105-6-100 PMC1127019

[98] Giot L Bader JS Brouwer C Chaudhuri A Kuang B Li Y Hao YL Ooi CE Godwin B Vitols E Vijayadamodar G Pochart P Machineni H Welsh M Kong Y Zerhusen B Malcolm R Varrone Z Collis A Minto M Burgess S McDaniel L Stimpson E Spriggs F Williams J Neurath K Ioime N Agee M Voss E Furtak K Renzulli R Aanensen N Carrolla S Bickelhaupt E Lazovatsky Y DaSilva A Zhong J Stanyon CA Finley RL Jr White KP Braverman M Jarvie T Gold S Leach M Knight J Shimkets RA McKenna MP Chant J Rothberg JM A protein interaction map of Drosophila melanogaster Science 2003 302 1727 1736 14605208 10.1126/science.1090289

